# Prioritization of treatment: need- or desire-based approach with a three-year follow-up

**DOI:** 10.1186/1746-160X-8-S1-P10

**Published:** 2012-05-25

**Authors:** D Divekar

**Affiliations:** 1 Independent consultant, Pune, India

## 

In general, medical teams do not prioritize treatment in a way to establish mental health prior to other parts of a treatment sequence. Patients with ectodermal dysplasia may not be compromised systemically, but mentally they surely are. In cases of ectodermal dysplasia, the psychological inadequacy may be attributed to the characteristic facial appearance and the absence of teeth. Such cases can be better managed by prioritizing treatment, choosing a desire-based approach rather than a need-based course of action. Improving the facial appearance first will strengthen the confidence of the patient in the operator and increase his overall acceptance in the society. Overcoming psychological barriers allows intra-oral rehabilitation with lesser effort and increases its success.

